# Using Preprints for Journal Clubs

**DOI:** 10.1128/mBio.00516-18

**Published:** 2018-04-03

**Authors:** Arturo Casadevall, Neil Gow

**Affiliations:** a Johns Hopkins Bloomberg School of Public Health, Baltimore, Maryland, USA; b University of Aberdeen, Aberdeen, United Kingdom

**Keywords:** journal club, preprints, publishing

## Abstract

Journal clubs are important mechanisms for teaching how to approach the scientific literature critically and for disseminating findings. Papers from high-impact journals often dominate journal club selections, a practice that reinforces the unscientific emphasis of placing high value on publishing venue rather than scientific content and critical analysis of the publications. We suggest improving journal clubs by including preprints rather than focusing completely on published papers. This change in practice might benefit the scientific enterprise in numerous ways, including by providing direct criticisms to preprint authors before publication, deemphasizing publishing venue, teaching students the art of reviewing papers, and making journal clubs more current by discussing unpublished data.

## EDITORIAL

A journal club is a meeting to discuss a scientific paper. The practice of journal clubs originated in medicine and provided a mechanism for physicians to discuss new findings in the field (1). Journal clubs are used in academia, research laboratories, industry, and government as mechanisms for keeping current in certain areas of science. In academia, such clubs serve the essential role of providing a venue for instructing students on how to review the literature critically as well as extend awareness of topics of interest.

In a typical journal club, the presenter provides a detailed description of the paper. The goal is to show trainees how to read the literature critically and to discuss new information. A nonscientific survey of immunology and microbiology journal club publication choices at four universities found in open Internet websites provided some insight into journal choices. Of 112 journal club publication choices, 30 (27%) were from *Cell*, *Nature*, or *Science*. When these journals and their offshoots published by Cell Press and Nature Publishing Group (e.g., *Immunity*, *Nature Immunology*, etc.) were added, the total was 59 (53%). Journals published by scientific societies, such as those published by the American Society for Microbiology (such as *mBio*), the National Academy of Sciences (*Proceedings of the National Academy of Sciences of the United States of America*), and the American Association of Immunologists (*Journal of Immunology*), were a minority, accounting for less than 30% of journal selections.

In our opinion, the quality of journal clubs has been degraded in recent years as another deleterious consequence of the impact factor mania that has gripped the biological sciences (2). Specifically, presenters often feel under great pressure to pick leading articles to avoid criticism about their selections. This frequently leads to choosing publications from very high impact journals. Articles in these journals tend to be short and terse and have a greatly abbreviated methods section. Consequently, critical details of how the work was performed may not be available for discussion. In this situation, the conversation frequently digresses into a discussion of how and why the paper made it into such a journal, with a tacit assumption that all important critical analysis has been done via high-level peer review. Such discussions tend to miss the science and the objective of acquiring critical skills, and when that happens, it diminishes the opportunities for teaching and learning. Previous editorials have recommended that journal clubs broaden their selections to include papers from more-specialized journals to avoid these problems (2).

In recent years, the biological sciences have experienced the phenomenon of the preprint movement (3–5). Although the circulation of unpublished papers has long been used by other disciplines, the notion of depositing unpublished papers in preprint servers that are available to all free of charge is new to biology. Preprint servers, such as Biorxiv, screen submissions and accept only those items that pass muster as scientific papers. Most submissions are freely available to all days after the item is placed in the preprint server. These preprint servers include boxes where readers can enter comments on the papers, thus allowing for prepublication criticism and crowd source reviewing. The practice is increasingly popular, and the number of manuscripts deposited in Biorxiv is growing exponentially (http://asapbio.org/preprint-info/biology-preprints-over-time).

There are numerous reasons why using preprints for journal clubs instead of published articles might improve that experience while also benefiting the scientific enterprise. (i) Preprints are free and available to all with an Internet connection. This might be a major advantage in resource-poor settings where the selected article is behind a pay wall and not accessible to all. Although such costs are often minimized by distributing the article to journal club members, that practice can run afoul of restrictions by certain publishers. (ii) Preprints are often the most current form of scientific information since they are deposited before the article is published. Given that publication of a manuscript can be associated with significant delay as a result of peer review and publication procedures, such as the generation and correction of article proofs, using preprints in journal clubs will heighten the immediacy of the information. (iii) Presenting preprints will give the journal club members the ability to participate in peer review, since comments and criticisms generated during the discussion can be posted on preprint servers. For preprints deposited in the Biorxiv server, there is a built-in commenting service that allows the comments to be directly linked to the manuscript. Having more eyes look at each manuscript might improve the literature by reducing the frequency of inappropriately reproduced images, which plagues up to 4% of all published articles (6). Hence, the journal club review might not only teach how to approach the literature critically but also actually make a difference in the quality of research that is published. (iv) Using preprints deemphasizes the current focus on selecting articles from high-impact journals. This will change the focus from publication venue to focusing on the content of the paper and might improve the overall scientific discussion and sharpen the ability of students to develop their own view of what constitutes rigorous and effective experimentation and communication. (v) Preprints might allow a later analysis of the work by comparing the preprint with the final paper. This type of comparison would allow students and scientists to see the effect of the peer review process on publication, and that would provide additional opportunities for learning.

In summary, we see the introduction of preprints into journal club choices as a positive step that might improve the scientific enterprise at many levels, from providing additional means for prepublication criticism to refocusing the discussion to the scientific issue of the work. The use of preprints in journal clubs was previously suggested by others, who argued for some of the same benefits noted above. A Web platform to facilitate peer review of preprints is already available (http://asapbio.org/preprint-journal-clubs). In this editorial, we strongly endorse those suggestions. Institutions that adapt this practice will further empower the preprint movement.

## References

[1] Linzer M 1987 The journal club and medical education: over one hundred years of unrecorded history. Postgrad Med J 63:475–478. doi:10.1136/pgmj.63.740.475. 3324090 PMC2428317

[2] Casadevall A, Fang FC 2015 Impacted science: impact is not importance. mBio 6:e01593-15. doi:10.1128/mBio.01593-15. 26463169 PMC4620476

[3] Schloss PD 2017 Preprinting microbiology. mBio 8:e00438-17. doi:10.1128/mBio.00438-17. 28536284 PMC5442452

[4] Bourne PE, Polka JK, Vale RD, Kiley R 2017 Ten simple rules to consider regarding preprint submission. PLoS Comput Biol 13:e1005473. doi:10.1371/journal.pcbi.1005473. 28472041 PMC5417409

[5] Vale RD 2015 Accelerating scientific publication in biology. Proc Natl Acad Sci U S A 112:13439–13446. doi:10.1073/pnas.1511912112. 26508643 PMC4640799

[6] Bik EM, Casadevall A, Fang FC 2016 The prevalence of inappropriate image duplication in biomedical research publications. mBio 7:e00809-16. doi:10.1128/mBio.00809-16. 27273827 PMC4941872

